# Activated sludge microbial community assembly: the role of influent microbial community immigration

**DOI:** 10.1128/aem.00598-24

**Published:** 2024-07-12

**Authors:** Claire Gibson, Shameem Jauffur, Bing Guo, Dominic Frigon

**Affiliations:** 1 Department of Civil Engineering and Applied Mechanics, McGill University, Montreal, Quebec, Canada; 2 Department of Civil and Environmental Engineering, Center for Environmental Health and Engineering, University of Surrey, Surrey, United Kingdom; University of Michigan, Ann Arbor, Michigan, USA

**Keywords:** microbial community assembly, immigration, activated sludge, wastewater treatment, synthetic wastewater

## Abstract

Wastewater treatment plants (WWTPs) are host to diverse microbial communities and receive a constant influx of microbes from influent wastewater. However, the impact of immigrants on the structure and activities of the activated sludge (AS) microbial community remains unclear. To gain insight on this phenomenon known as perpetual community coalescence, the current study utilized controlled manipulative experiments that decoupled the influent wastewater composition from the microbial populations to reveal the fundamental mechanisms involved in immigration between sewers and AS-WWTP. The immigration dynamics of heterotrophs were analyzed by harvesting wastewater biomass solids from three different sewer systems and adding to synthetic wastewater. Immigrating influent populations were observed to contribute up to 14% of the sequencing reads in the AS. By modeling the net growth rate of taxa, it was revealed that immigrants primarily exhibited low or negative net growth rates. By developing a protocol to reproducibly grow AS-WWTP communities in the lab, we have laid down the foundational principles for the testing of operational factors creating community variations with low noise and appropriate replication. Understanding the processes that drive microbial community diversity and assembly is a key question in microbial ecology. In the future, this knowledge can be used to manipulate the structure of microbial communities and improve system performance in WWTPs.

**IMPORTANCE:**

In biological wastewater treatment processes, the microbial community composition is essential in the performance and stability of the system. This study developed a reproducible protocol to investigate the impact of influent immigration (or perpetual coalescence of the sewer and activated sludge communities) with appropriate reproducibility and controls, allowing intrinsic definitions of core and immigrant populations to be established. The method developed herein will allow sequential manipulative experiments to be performed to test specific hypothesis and optimize wastewater treatment processes to meet new treatment goals.

## INTRODUCTION

In activated sludge wastewater treatment plants (AS-WWTPs), the microbial community composition is intricately related to the performance and stability of the system. However, a comprehensive understanding of the structuring process of the community remains elusive. During the activated sludge (AS) treatment process, a diverse heterotrophic microbial community is grown in the plant to become highly effective at degrading the organic pollutants contained in the incoming wastewater. In recent years, efforts have been made to modify these processes to improve performance and meet new objectives for wastewater management. Modifications often require control over specific functional populations within the diverse AS microbial community. For example, enhanced phosphate removal processes require the growth of Polyphosphate Accumulating Organisms (e.g., members of the genera *Candidatus* Accumulibacter and *Tetrasphera*) (1), whilst to curtail operational problems, a reduction in the abundance of detrimental bulking and foaming bacteria (e.g., genera *Gordonia* and *Thauera*) is desired (2). With a limited knowledge of community assembly mechanisms, modification and optimization of the AS process is challenging. A greater understanding of the underlying microbial mechanisms involved in community assembly and maintenance is crucial to benefit the engineering and function of these systems.

Previous studies on microbial community assembly focused on the impact of temporal differences (3), wastewater characteristics (4), operational conditions (5, 6), and geographical location (7) on the AS community. However, the role of the diverse and continuously immigrating influent microbial community is poorly understood. This is a special case of community coalescence with a perpetual occurrence which likely drives in part the community dynamics (8).

The community in municipal wastewater originates from numerous environments including human feces, sewer biofilm and sediments, and soil runoffs (3). Studies have produced conflicting results on the relative importance of influent immigration on the assembly of the AS community. Some suggested that the overall effect of the influent community is negligible, as they observed that the AS microbial community remains stable over time despite changes in the composition of the influent wastewater community (4). The activity of immigrating taxa has also been questioned, with some studies showing these populations often have low or negative net growth rates (9, 10).

Other literature reports argued influent immigration to be an important process in microbial community assembly. In lab-scale AS reactors operated at extremely low temperatures and solid retention times (SRTs), immigration from sewers was found to be essential to maintain complete nitrification (11). The occurrence of shared operational taxonomic units (OTUs) between influent and AS communities also infer high immigration rates in full-scale WWTPs (9, 12). Furthermore, a recent study of 11 AS-WWTPs in Denmark, selected based on their highly similar process design, identified the AS community composition to be strongly influenced by the influent wastewater community composition (13). However, it remains difficult to ascertain if the influent community composition itself is an influencing factor independent of the substrate composition of the wastewater.

With contrasting reports on the impact of immigration, it is challenging to understand the relative importance of this process. This variability can be somewhat explained by the limited methods used to study the immigration process, which lack reproducibility and assessment of the activity of immigrants. As reviewed by Mei and Lie (14), commonly used approaches include counting shared microbial species between ecosystems (i.e., the influent and WWTP), microbial source tracking, and neutral community modeling. However, these approaches only imply a contribution from the immigrating source and provide little information about the fate and activity of these organisms. Furthermore, neutral modeling approaches to investigate immigration (such as the neutral theory of biodiversity) assume that organisms are functionally equivalent (15); this assumption is likely inaccurate considering the variety of substrates available to the heterotrophic populations (16) and the niche differences between the influent and AS. It has been proposed that immigrants should be classified as either rare diffusive immigrants or time continuous high-rate mass-flow immigrants (17). Heterotrophic mass-flow immigrants appear to be heavily influenced by deterministic selection, suggesting that they should be divided into relevant functional guilds when assessing their assembly mechanisms. Consequently, we cannot rely on mathematical modeling approaches alone to resolve the relative importance of selection, immigration, and drifts in the assembly of the community. Furthermore, full-scale systems display too much variability in terms of wastewater composition, operation, and community to allow appropriate reproducibility and provide an accurate assessment of the mechanisms at play during immigration through manipulated experiments.

Given the complex behavior of microbial communities within wastewater, reproducible and highly controlled systems are required to accurately investigate and quantify the impact of immigration into WWTPs and determine the fate of immigrating bacteria. Because the presence of specialized populations in the influent wastewater is often correlated with the presence of specific substrates (18), it is impossible to understand immigration independently from the wastewater substrate compositions in full-scale systems. Therefore, here, we propose the development of a system where the substrate landscape and the immigrating community can be manipulated independently using highly controlled laboratory-scale reactors to investigate fundamental questions on the quantitative impact of immigration and activity with appropriate reproducibility and controls. Using a series of highly controlled reactors (a total of 72 reactors were operated for 17–25 weeks), we manipulated the influent wastewater composition and microbial content independently to identify the effect of constant immigration on the AS microbial community. The laboratory-scale AS reactors were inoculated with activated sludge samples taken from three AS-WWTPs, representing three of the most different municipal AS microbial communities among the WWTPs located within 100 km of Montréal (Québec, Canada). The purpose of this was to determine whether the impact of immigration was dependent on the established community within the reactors. This also validated the reactor protocol for future use with different but similar starting communities. Finally, dynamics in the AS microbial community compositions were analyzed using 16S rRNA gene amplicon sequencing. The long-term goal of the current work is to provide a reproducible protocol to conduct studies on the fate of immigrants and their activity in AS microbial communities.

## RESULTS

### Variations in overall community compositions during the experiment

**Fig 1:**
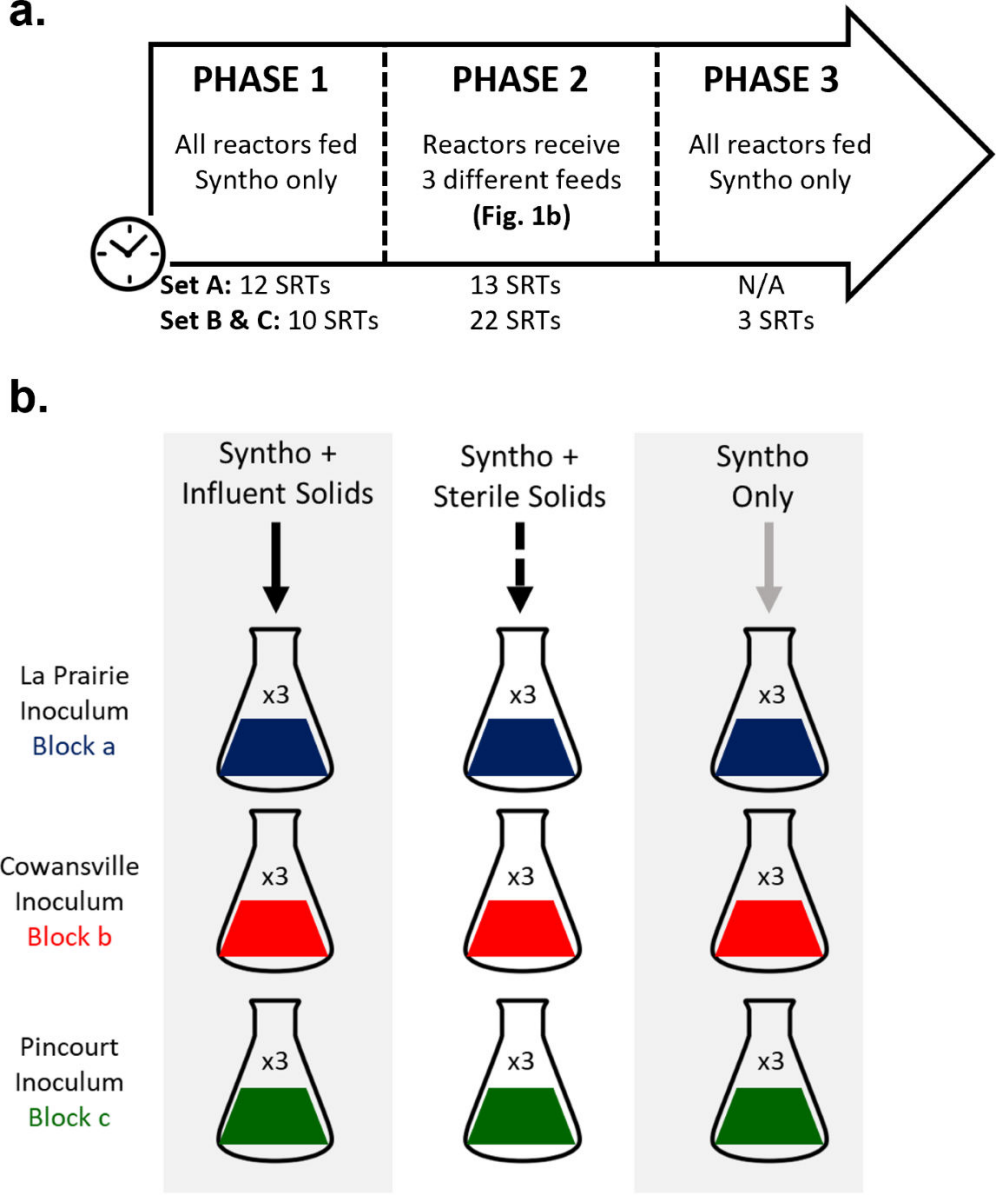
Experimental set up. (**a**) Schematic of one set of reactors (i.e., receiving one source of influent solids) in Phase 2. Three sets of reactors were operated in total. Set A received La Prairie influent solids; Set B, Cowansville influent solids; Set C, Pincourt influent solids. The block describes the inoculum-activated sludge-mixed liquor source. (**b**) Timeline of reactor operation. Reactors were operated in three phases with feed altered.

Determining the specific impact of influent immigration on the structure of the AS community in full-scale systems presents a significant challenge because of the numerous spatial and temporal variations between locations. The current study utilized controlled reactor experiments to investigate the impact of immigration on a naturally assembled, diverse microbial community. As shown in Fig. 1a, during Phase 1, all reactors received Syntho [a synthetic wastewater (19)] to develop a steady-state core microbial community adapted to a specific substrate composition. This procedure also minimized the biomass of *residual immigrants* (immigrant populations not consuming resources as defined in section Definitions of Population Categories) from the inocula remaining in the system prior to the test period (i.e., Phase 2).

**Fig 2:**
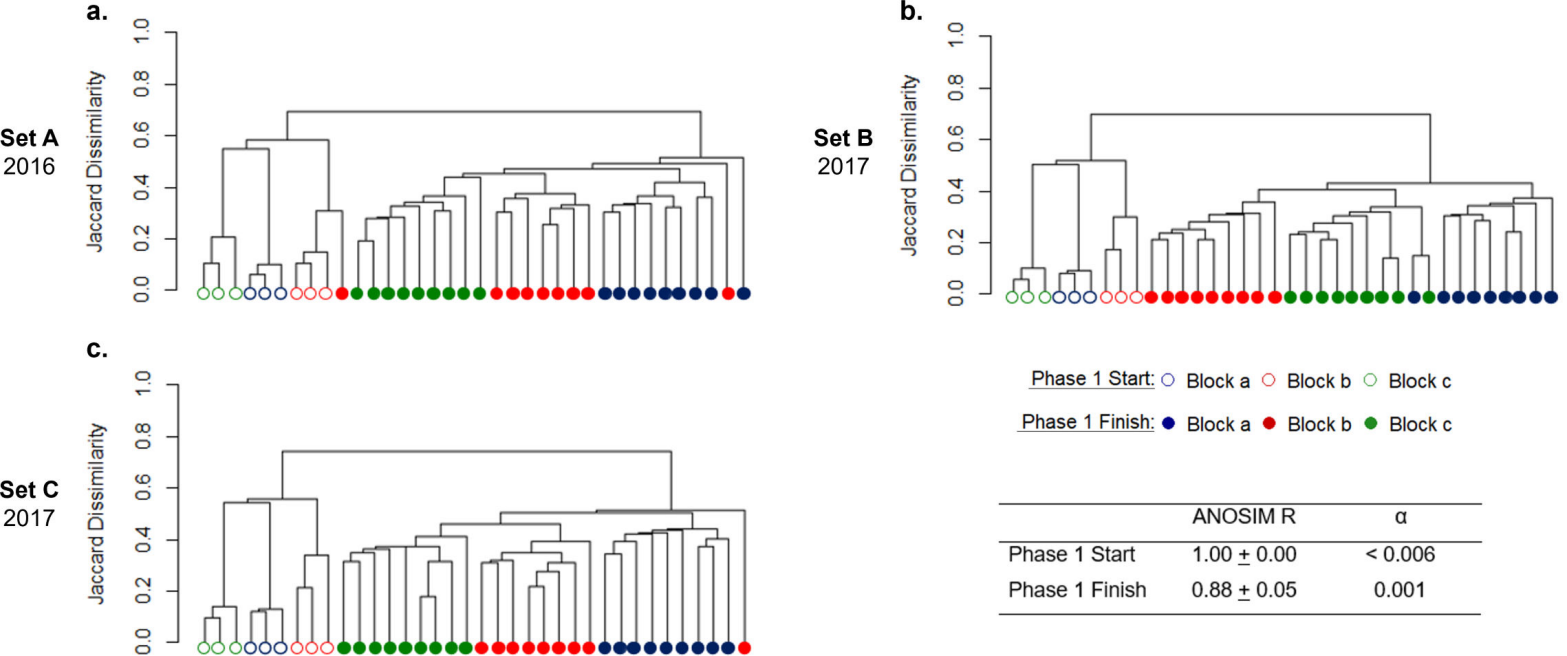
Tree dendrogram of the microbial community at the start (hollow symbols) and end (filled symbols) of Phase 1 using UPGMA clustering method and the Jaccard dissimilarity. Set A (**a**) was operated in 2016, whilst Sets B (**b**) and C (**c**) were operated in 2017. The Block indicates the source of inoculum: Block a- La Prairie activated sludge, Block b- Cowansville activated sludge, and Block c- Pincourt activated sludge. Inoculum communities were the same for Sets B and C as they were operated in tandem. Each inoculum (hollow symbols) represents a single biological sample that was extracted and sequenced three times, whilst the end of Phase 1 (filled symbols) are communities grown in independent reactors.

During Phase 1 when fed with Syntho only, the microbial communities of the reactors became more similar to one another when compared to the differences among starting inocula (Fig. 2), and the amplicon sequencing variant (ASV) richness decreased (Fig. S1). Irrespective of the inoculum, 46.2% ± 4.7% (± indicates SD) of the observed genera at the end of Phase 1 were shared between the 27 resulting AS reactor communities forming the *core resident populations* of Syntho (as defined in section Definitions of Population Categories), whilst at the beginning of Phase 1, only 27.1% ± 4.7% of the observed genera were shared among the three inocula. The ANalysis Of SIMilarity (ANOSIM) can be used to express in a common quantity the differences between clusters, with ANOSIM R values closer to 1.0 indicative of higher dissimilarity between groups, whilst those closer to 0.0 indicate higher similarity. Between the start and end of Phase 1, the ANOSIM R reduced from 1.00 to 0.88 indicating higher similarity between the reactor microbial communities. Nonetheless, the majority of AS reactor communities at the end of Phase 1 remained significantly clustered based on the inoculum received (Fig. 2: Jaccard dissimilarity, Fig. S2: Bray-Curtis dissimilarity). As the experiment progressed, the microbial community of most reactors receiving the same feed remained clustered based on the inoculum received (Table S3) with the exception of a few outliers as visualized in Fig. S1.

**Fig 3:**
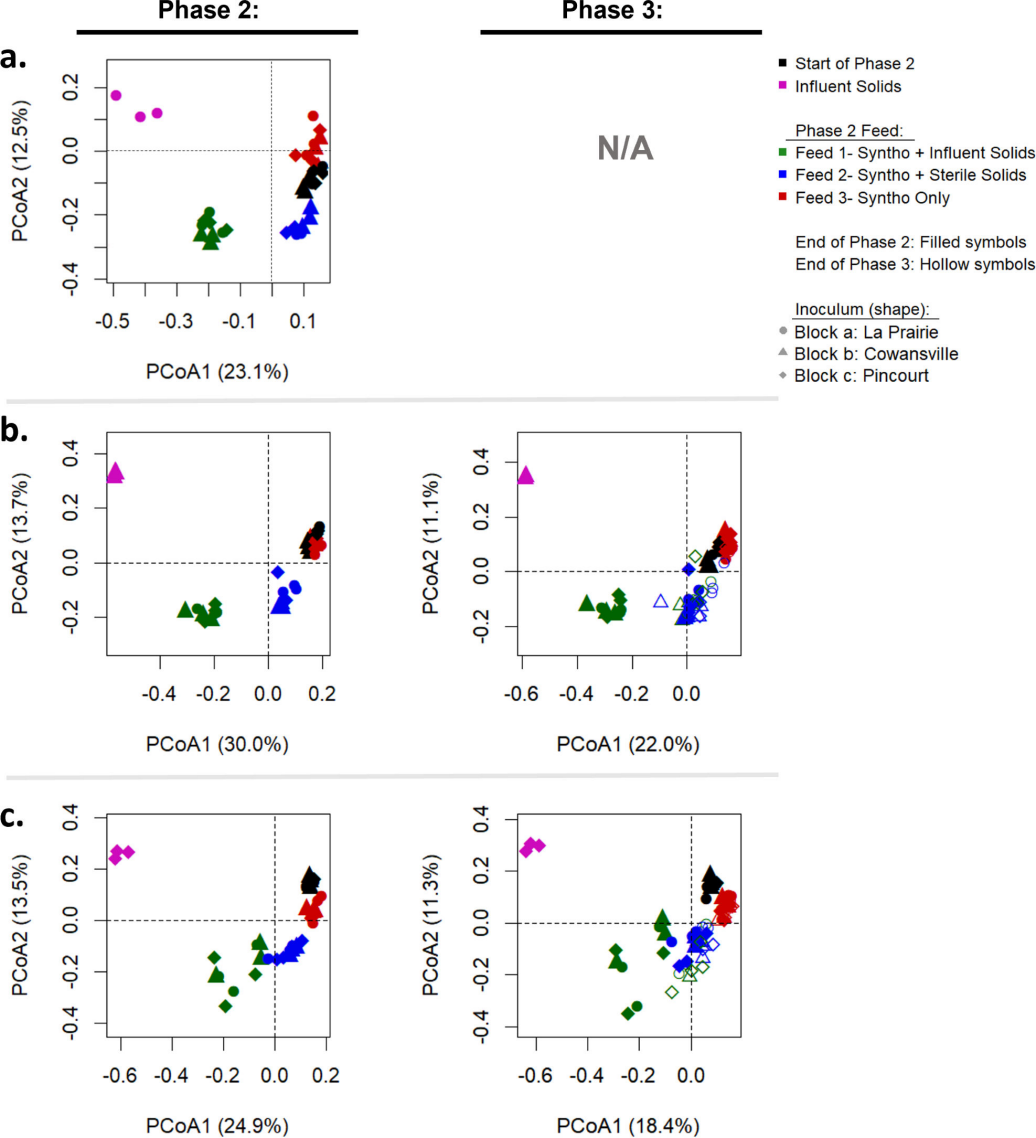
Principal coordinate analysis of reactors communities at the end of Phases 2 and 3, visualized at ASV level using Jaccard dissimilarity. (**a**) Set A- La Prairie Influent Solids. (**b**) Set B- Cowansville influent solids. (**c**) Set C- Pincourt influent solids. Filled symbols represent samples taken at the end of Phase 2, and hollow symbols were samples collected at the end of Phase 3.

During Phase 2, reactors received three different feeds. The test reactors received a feed comprising of Syntho supplemented with influent solids to determine the impact of immigration. The remaining reactors received Syntho and sterile influent solids or Syntho only, which acted as substrate and continuity controls, respectively. At the end of Phase 2, principal coordinate analysis (Fig. 3) revealed that immigration caused the reactor AS communities to become more similar to the influent community, as shown by the points representing these communities being located closer to the influent community along the PCoA1 axis. The microbial community of the reactors receiving sterile solids also moved away from the starting position, likely due to the additional substrate and residual DNA in the autoclaved influent biomass (Fig. 3; Fig. S3: Bray-Curtis Dissimilarity).

During Phase 2, the ASV richness of reactor Set A (test reactors receiving La Prairie influent solids) and B (test reactors receiving Cowansville solids) significantly increased (Fig. S4; *P* < 0.001 and 0.0001, respectively). The impact of immigration on the richness of reactor Set C (receiving Pincourt influent solids) was more variable (*P* value not significant). This could be associated with differences in the niche availability in these reactors due to drift in the microbial community composition over time. In reactor Set A, reactors receiving autoclaved influent solids also showed a slight but significant increase in richness (*P* > 0.001), likely due to the introduction of residual DNA remaining after sterilization. Reactors receiving Syntho only throughout either showed no significant change or a slight reduction in richness during Phase 2 (Set A: NS, Set B: *P* = 0.0002, and Set C: *P* = 0.04). The original richness of the reactors was not restored to that of the starting community (Fig. S4; P1S), likely due to the relative simplicity of the substrate-composition of Syntho compared to actual wastewater and the greater homogeneity of laboratory-scale flask reactors than full-scale WWTPs.

Phase 3 of reactor operation was introduced to determine whether the impact observed with immigration could be sustained over time without continuous seeding of influent solids. During this phase, influent solids were removed from the feed, and all reactors received Syntho only as in Phase 1. Within three SRTs (where 1 SRT is 5 days), the communities in reactors that previously received active influent solids during Phase 2 moved back toward their starting position (Fig. 3), becoming more similar to the reactors that received Syntho only throughout. At the end of Phase 2, the ANOSIM R values between the reactors receiving influent solids and those receiving Syntho only throughout were 0.92 and 0.49 (*P* < 0.001) in the Cowansville and Pincourt reactors, respectively, indicating a high degree of dissimilarity. However, by the end of Phase 3, the communities became more similar with the ANOSIM R reduced to 0.36 in the Cowansville reactors (*P* < 0.01) and 0.28 in the Pincourt reactors (*P* = 0.001). These results suggest that the full impact of immigration could not be maintained over time without continuous seeding.

### Classification of genera

To provide a more detailed analysis of the impact of immigrating taxa, the genera forming the microbial community at the end of Phase 2 were classified into four categories according to their presence in reactors receiving influent solids. This analysis was conducted at the genus level as ASVs within the same genus are likely to have similar ecological functions and because direct comparisons of ASVs between the influents and the AS were often quantitatively unreliable due to the limited sequencing depth and their low abundances in one of the two compartments. The four categories assigned are outlined in greater detail in the Definitions of Population Categories section and are briefly recalled here. First, a genus was defined as a *core resident population* if it occurred in at least 80% of all the reactors within a set (with the same influent solids source) with a relative abundance of at least 0.1%. Second, *non-core resident genera* appeared in reactors without immigration but in fewer than 80% of these reactors or at an average abundance below 0.1%. Third, *growing immigrant genera* were only present in the reactors receiving influent solids and were at a higher abundance than in the reactors receiving autoclaved solids in the same set (i.e., same influent solids source). These genera were presumed to be actively growing. Finally, *residual immigrant genera* were those present only in the reactors receiving influent solids and occurring in the reactors with immigration in equal or lower abundance to the sterile autoclaved control group within the same set.

The resident community in all reactors consisted of between 50 and 73 genera depending on the set of reactors (i.e., the source of the influent community), and these genera accounted for between 69% and 76% of the total reads observed in the reactor set (Table 1). Conversely, the immigrant populations showed a higher diversity (118–206 genera) but accounted for much fewer reads from the reactor set (4%–14%). Therefore, operation and the Syntho substrate composition appeared to have determined the resident abundant members of the communities, whilst immigration contributed toward low abundance members of the communities.

**TABLE 1:** Percentage of overall reads which form the resident and immigrant communities at the end of Phase 2 for reactor sets receiving different influent solids

	Set A: La Prairie		Set B: Cowansville		Set C: Pincourt	
	Genera^*a*^	Reads (%)^*b*^	Genera	Reads (%)	Genera	Reads (%)
Core residents	73	75.3 ± 5.4	54	76.0 ± 4.9	50	69.3 ± 11.5
Non-core residents	133	15.7 ± 4.3	121	7.9 ± 1.7	122	22.4 ± 12.8
Growing immigrants	127	8.2 ± 2.0	206	14.3 ± 4.9	118	4.2 ± 1.4
Residual immigrants	7	0.3 ± 0.2	5	2.1 ± 1.6	12	2.9 ± 3.2

^*a*^ Number of genera within the category.

^*b*^ Percentage of overall reads +SD.

**Fig 4:**
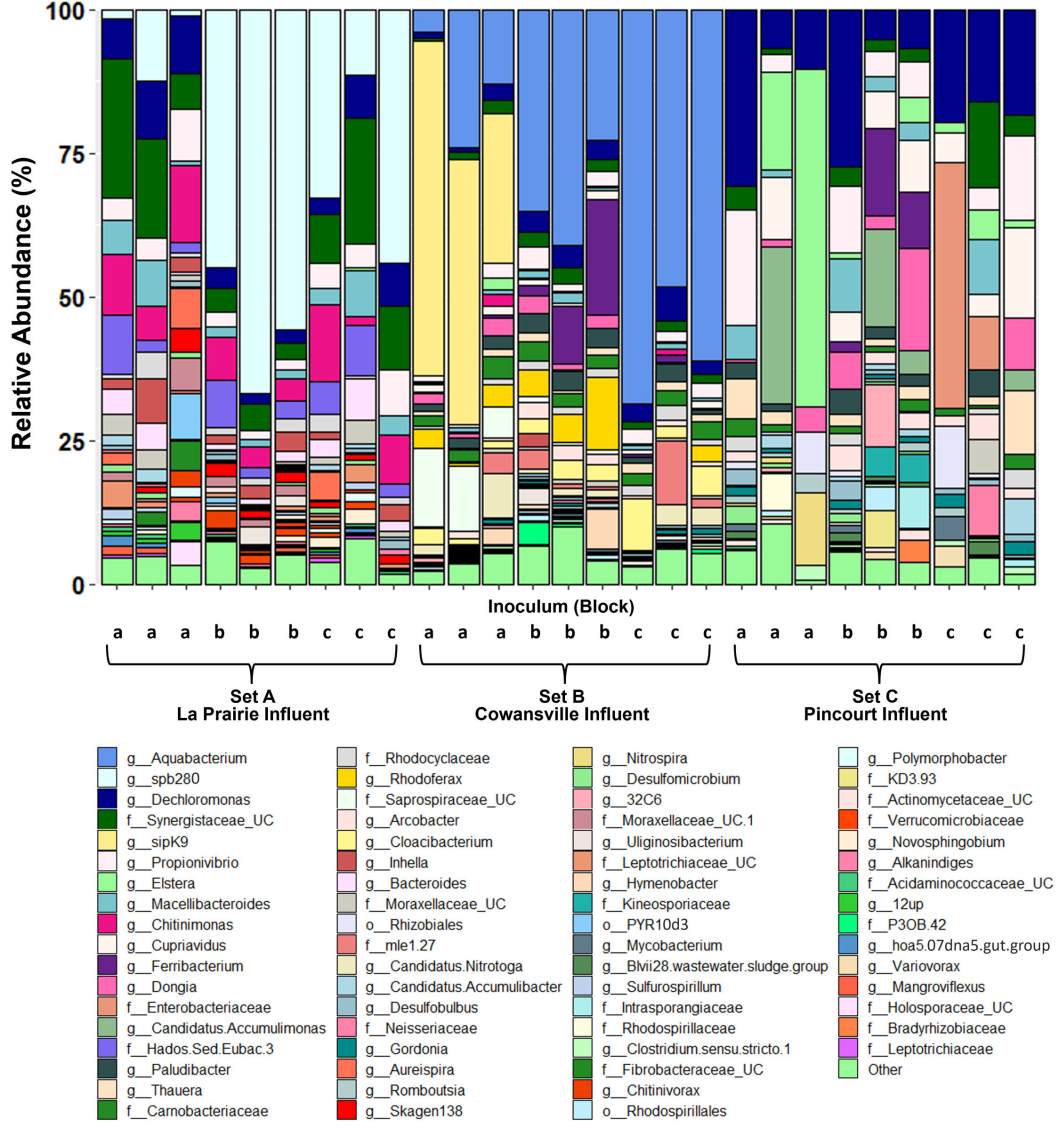
Top 70 immigrating genera, normalized to total immigrants per reactor. Inoculum a- La Prairie AS, b- Cowansville AS, and c- Pincourt AS. Set A- La Prairie influent solids, B- Cowansville influent solids, and C- Pincourt influent solids. UC in the legend indicates uncultured strains of a given taxa.

The immigrant population varied based upon the source of influent solids (Fig. 4). In reactor Set A, which received influent solids from La Prairie WWTP, the genus *Spb280* [genus midas_g_81, family *Comamonadaceae* (20)] and an uncultured genus of the family *Synergistaceae* were the most abundant immigrants. Whilst in Set B, members of the genus *Aquabacterium* (family *Comamonadaceae*) had the highest abundance, and in Set C, the most abundant immigrant genus was *Dechloromonas* (family *Rhodocyclaceae*). In addition to unique immigrant communities based on the source of influent solids, there also appeared to be some inoculum effect (between Blocks). Within the immigrant population of Set B reactors (receiving Cowansville influent solids), those that were inoculated with La Prairie activated sludge (Block a) were abundant in the genus SipK9 [genera *midas_g_1719 and midas_g_2835*, family *Rhodobacteraceae* (20)], accounting for up to 11.7% of the reads and explaining some of the variability in percentage reads contributed though immigration reported in Table 2. Whilst in the other Set B reactors inoculated with Cowansville and Pincourt activated sludge (Block b and c, respectively), SipK9 was not observed with the same high abundance. The function of SipK9 is currently unknown, but its abundance in the reactors may warrant further investigation.

**TABLE 2:** Percentage of overall reads which form the resident and immigrant communities at the end of Phase 3

	Set B		Set C	
	Cowansville influent		Pincourt influent	
	Genera^*a*^	Reads (%)^*b*^	Genera	Reads (%)
Core residents	54	85.7 ± 5.9	50	78.3 ± 15.2
Non-core residents	124	10.4 ± 5.0	121	19.4 ± 13.4
Growing immigrants	65	3.5 ± 2.1	29	1.8 ± 2.7
Residual immigrants	4	0.1 ± 0.1	9	0.2 ± 0.3

^*a*^ Number of genera within the category.

^*b*^ Percentage of overall reads +SD.

During Phase 3, influent solids were removed, and all reactors received Syntho Only as in Phase 1 to determine if immigration had a lasting effect on the microbial community. Phase 3 was only conducted for reactor Set B and C in 2017. At the end of Phase 3, the number of immigrants remaining in the reactors had reduced in both reactors Sets B and C (Table 2). In reactor Set C, immigrants remaining included taxa from the families *Rhodospirillaceae*, *Rhodocyclaceae*, *Nitrospiraceae*, and *Flavobacteriaceae*. In reactor Set B, the families *Rhodocyclaceae*, *Gallionellaceae* (genus *Candidatus* Nitrotoga), *Comamonadaceae* (genus *Rhodoferax*), and *Xanthomonadaceae* (genus sipK9) were among the most abundant growing immigrants remaining. To further investigate factors influencing the persistence of immigrants in the reactors at the end of Phase 3, further analysis of the net growth rate was conducted.

### Assessing the growth rate of genera

**Fig 5:**
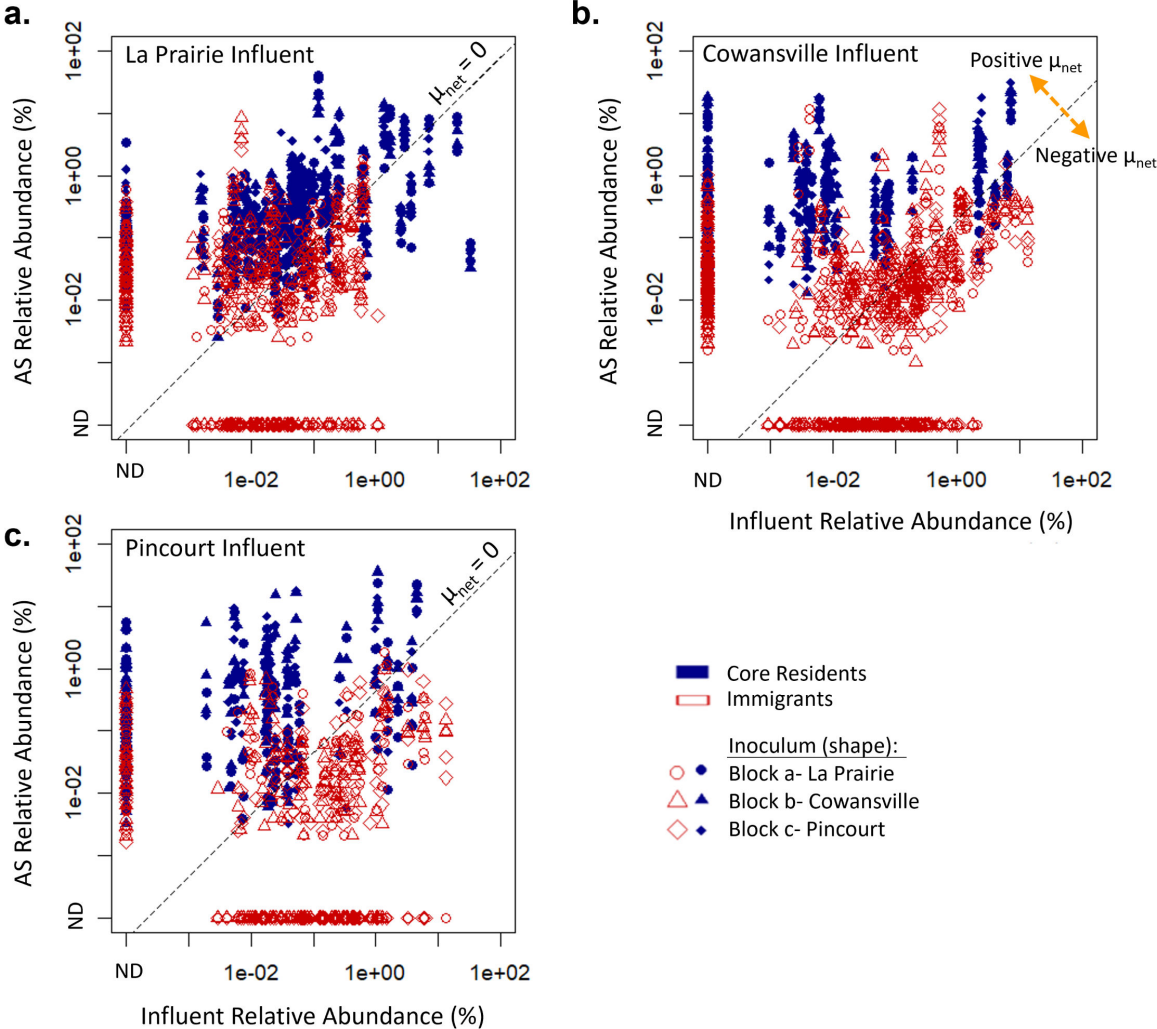
Comparison of relative abundance of immigrant and core resident genera in the influent and AS. “ND”: not detected indicates genera that were below the detection limit. Reactors received influent solids from different sources: (**a**) Set A- La Prairie influent solids, (**b**) Set B- Cowansville influent solids, and (**c**) Set C- Pincourt. The dashed line represents a net growth rate of 0. Genera on the same 45°-lines have the same net growth rates ($\mu_{net}$).

The impact of immigration on the various genera can be visualized with a log-log scatter plot of their relative abundances in the AS and influent microbial communities (Fig. 5). Assuming that the ASV composition of each genera is the same in the influent and in the AS, the log-log scatter plot of the relative abundances of the genera can be understood as representing their net growth rate by using a mass balance model (eq. 4). Based on the model in eq. 4, genera with different relative abundances but falling on the same 45°-lines on Fig. 5 have the same net growth rate. As a reference, a 45°-line was drawn on the scatter plots of Fig. 5 for net growth rates ($\mu_{net}$) equal to 0 (i.e., the point where the growth rate of the organisms is equal to its decay rate). Any points falling above the zero net growth rate line ($\mu_{net}=0$) display a positive net growth rate, whilst those below this line have an overall negative net growth rate. According to reactor theory, organisms displaying positive net growth rates should be maintained in the reactor without immigration assuming no other factors such as competition occur, whilst organisms displaying negative net growth rates would be washed out if immigration were stopped.

Genera classified in the core resident population by our criteria typically exhibited a positive net growth rate (Fig. 5), whilst the growing immigrant population typically had a negative net growth rate, particularly those from Cowansville and Pincourt influents (Set B and C; Fig. 5b and c). The selection was also observed, with some genera that were present in the influent at high relative abundance (up to 1% of the reads) remaining undetected in the AS [Fig. 5; shown as “ND” on activated sludge (AS) axis]. Interestingly, some genera classified as immigrants were not detected (ND) in the influent, suggesting that they occurred below the detection limit in the influent solids and increased to detectable levels within the reactor communities.

**Fig 6:**
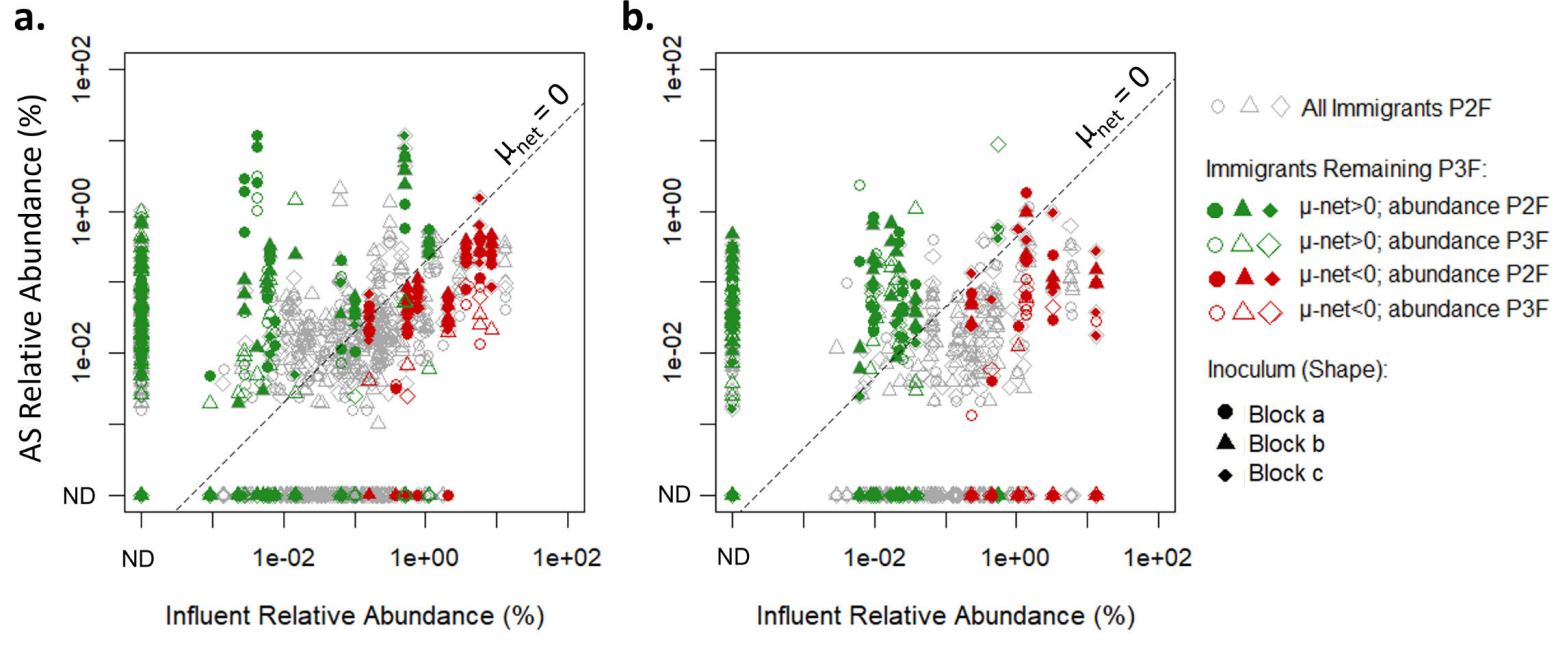
Comparison of growing immigrant genera at the end of Phase 2 (P2F) and Phase 3 (P3F). “ND”: not detected indicated genera which were below the detection limit. (**a**) Reactor Set B received Cowansville Influent Solids. (**b**) Reactor Set C received Pincourt Influent Solids. µnet was modeled as described in the section “Modeling net growth rate of bacteria” and Table S3.

In Fig. 5, it was observed that a selection of immigrants displayed a positive net growth rate. Based on reactor theory, it would be expected that these genera could be maintained within the reactors without continuous immigration. At the end of Phase 3, although reduced, a number of immigrants remained in the reactors (Table 2). Analysis of the net growth rate of these taxa (Fig. 6) showed that of those remaining at the end of Phase 3 between 75% and 77% had displayed an overall positive net growth rate during Phase 2. However, many other immigrants that had a positive net growth rate during Phase 2 were not detected at the end of Phase 3. An odds ratio test of the taxa remaining at the end of Phase 3 and their associated net growth rate evaluated in Phase 2 supported that taxa with a positive net growth rate were between 5.6 and 7.4 times more likely to persist at the end of Phase 3 than those with a negative net growth rate.

The remainder of the immigrants present at the end of Phase 3 was classified as having a negative net growth rate (Fig. 6). The detection of these taxa at the end of Phase 3 was unexpected; however, it was noted that these genera typically occurred in higher abundance at the end of Phase 2. During Phase 3, the abundance typically reduced by at least 85% suggesting that washout would likely occur over time. When excluding these taxa, an odds ratio test showed that taxa with a positive net growth rate were 14.0–15.2 times more likely to persist at the end of Phase 3 than those with a negative net growth rate. Other factors, such as the influent solids as an additional food source, also likely influenced the persistence or loss of these taxa during Phase 3.

### Impact of immigration on core resident genera

The quantitative assessment of immigration reported thus far focused exclusively on immigration-dependent populations, i.e., those that are introduced into the system and only present with continuous immigration. The core resident genera were present under all reactor conditions, both with and without immigration, and accounted for up to 76% of sequencing reads (Table 1). It was noted that the core resident genera were often detected within the microbial community of the influent wastewater; however, the ASVs observed often varied. Thus, it was hypothesized that immigration may contribute to the diversity of these genera at ASV level, although the core resident population remains relatively stable over time in all reactors irrespective of feed received.

**Fig 7:**
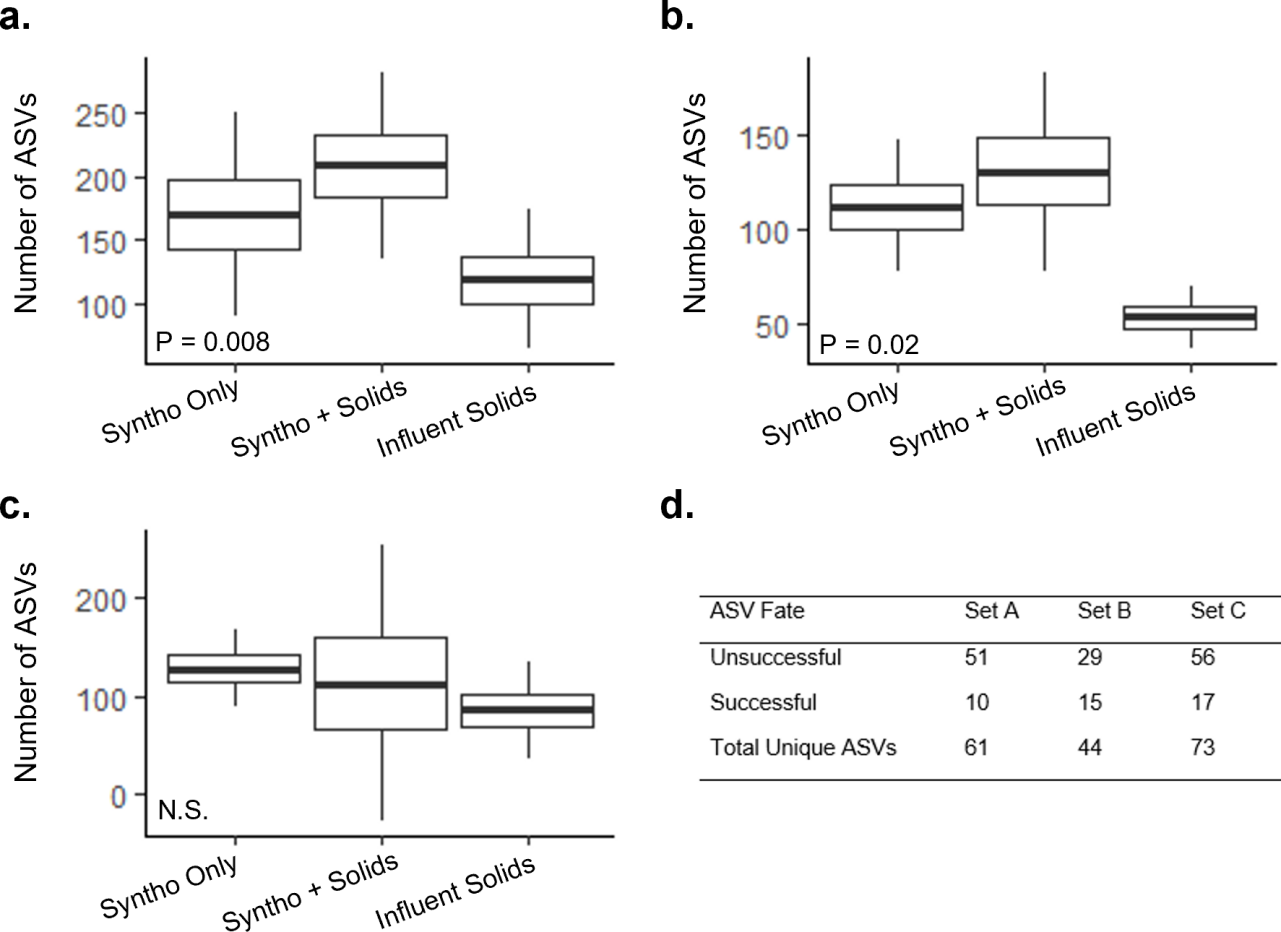
Impact of immigration on ASV richness of the core resident genera. (**a**) Reactor Set A received La Prairie influent solids, (**b**) reactor Set B received Cowansville influent solids, and (**c**) reactor Set C received Pincourt influent solids. (**d**) The fate of unique influent ASVs in the reactors; “unsuccessful” were those detected in the Influent, but not in the reactors, and “successful” were those that were detected in the reactors with immigration.

Analysis of the richness of core resident genera at the end of Phase 2 (Fig. 7) showed a significant increase in ASV diversity with immigration in Sets A and B of the reactors (Fig. 7a and b; unpaired *t* test *P* = 0.008 and *P* = 0.02, respectively). A significant increase was not observed in Set C of the reactors (Fig. 7c), where the impact of immigration on ASV diversity appeared to be more variable. Overall, the influent microbial community contained between 44 and 73 unique ASVs that belonged to genera previously classified as core residents (Fig. 7d). The introduction of these ASVs with immigration resulted in an increase in the richness of the core resident population. However, it should be noted that the selection was observed, with only 10–17 of the unique influent ASVs detected in the reactors receiving influent solids. Of those successfully immigrating were ASVs from the genera *Acinetobacter*, *Pseudomonas*, and *Zoogloea*. The genus *Acinetobacter*, which was highly diverse without immigration (up to 15 ASVs), had three additional ASVs introduced. However, selection was also observed, and several *Acinetobacter* influent ASVs were not detected in the reactors. Multiple sequence alignment was used to study the sequence similarity between the ASVs (Fig. S5a), and it was observed that ASVs that formed the core *Acinetobacter* population and successful immigrants typically clustered together.

Similarly, up to four additional *Pseudomonas* ASVs were introduced with immigration, whilst other influent ASVs did not successfully immigrate. Multiple sequence alignment of the *Pseudomonas* ASVs did not show clear clustering based on the fate of a given ASV (Fig. S5b). In the genus *Zoogloea*, all influent ASVs were detected in the reactors with immigration. Multiple sequence alignment of the *Zoogloea* ASVs revealed no clustering based upon their occurrence in the core resident populations or influent solids (Fig. S5c).

## DISCUSSION

The importance of influent immigration has long been debated among technical professionals, theorists, and modelers alike. One factor obscuring this debate in studies of full-scale wastewater treatment plants is the impossibility to differentiate the impact of influent substrate landscape from the immigration of microbial populations. To achieve this differentiation, the current study aimed to develop a reproducible and controlled system to investigate the impact of immigration and its relative contribution to the wastewater treatment plant community. Substrate and continuity controls were included to determine the fate of immigrating bacteria, and three different inocula were used to assess the impact of the WWTP community itself.

### Wastewater substrate composition and operation cause communities to become more similar

The impact of wastewater substrate composition on the activated sludge community is well documented in the literature (21, 22). Thus, it was as expected that during Phase 1, the introduction of a common wastewater source and identical operational conditions caused the inoculum communities to become more similar (Fig. 2).

At the end of Phase 2, reactor Sets A and B remained significantly clustered based upon the inoculum received (Fig. S1; Table S3; Jaccard distance, ANOSIM R = 0.77 ± 0.13). However, in reactor Set C, clustering was not observed in the test reactors that received influent solids, suggesting that features of the starting community were impacted by immigration (Table S3).

On a larger scale, with an increase in the popularity of microbial transplantation research over recent years to improve feed efficiency in livestock (23) or to restore the function of commensal gut microbiota and reduce the prevalence of multidrug-resistant organisms (24), the plausibility of activated sludge bioaugmentation has been raised. This concept would involve taking a desired activated sludge microbial community from one location and transplanting it into a WWTP requiring optimization. The current study suggests that activated sludge bioaugmentation may have variable results, as the activated sludge community is largely dependent on the wastewater substrate composition and not the inoculum alone.

### Immigration impacted the microbial community of the activated sludge

Previous studies have inferred the impact of immigration based upon shared taxa between the influent and WWTP, which provides limited information about the microbial activity of these organisms within the WWTP. The inclusion of a sterile substrate control in this study allowed us to quantitatively assess the impact of immigration, enabling the active and inactive portions of the immigrating community to be distinguished experimentally. Actively growing immigration-dependent taxa were found to account for between 4% and 14% of the total reads in the AS, contributing a significant proportion of the community (Table 2). This result was consistent with previous studies that reported 10% of OTUs to be present primarily due to immigration (9). Considering the significant proportion of AS reads contributed through immigration, this source should not be neglected in process design and optimization. Nonetheless, due to the low abundance of immigrant populations, analysis at ASV level was not possible. Future immigration studies should consider performing deeper sequencing strategies to enable analysis of ASV diversity in taxa occurring at lower abundance.

Beyond the immigration-dependent genera, immigration also impacted the core resident populations (Table 1). It was observed that genera classified as core residents in the AS were often present in the influent wastewater, and the ASV composition differed between the two environments. As a result, the introduction of new ASVs with immigration caused a significant increase in the diversity of the core resident population in the AS in two of the three sets of reactors (reactor Set A and B; Fig. 7a and b). Overall, between 1% and 6% of the AS sequencing reads were contributed by immigrating ASVs belonging to genera classified as core residents. As a result, the steady-state model used in this study may somewhat inaccurately estimate the growth rate of some core resident genera receiving immigrant ASVs.

Selection was an important factor in filtering the immigrating populations (Fig. 7d). The majority of unique ASVs identified in the influent solids were not detected in the reactors with immigration. Overall, between only 16% and 34% of ASVs unique to the influent wastewater successfully immigrated into the reactors. This is consistent with previous studies that observed heterotrophs to be strongly selected between influent wastewater and the activated sludge (12). This is likely due to niche differences between the environments or competition for resources with the well-established, metabolically similar reactor core resident community. The influence of selection varied at the genus level among the resident core genera (Fig. S5). For example, ASV selection was observed in the genus *Acinetobacter* but not in the genus *Zoogloea*. This supports the proposal that heterotrophs should not be considered as a whole but instead divided into relevant functional guilds for proper dynamic analyses.

### Growth rate is a key determinant in the fate of immigrants

The analysis of the resident and immigrant populations (Fig. 5) showed differences in their net growth rates. Resident genera typically exhibited positive net growth rates, thus explaining how they are present without constant seeding. Based upon the terminology proposed by Frigon and Wells (17), the majority of immigrants could be classified as mass-flow immigrants (i.e., continuous immigration at a high rate), as they displayed low or negative net growth rates in the AS, particularly in reactor Sets B and C. This could be attributed to differences in niche availability between the influent wastewater and the reactor communities. These results are consistent with those predicted in studies using a mass balance approach, which showed a large proportion of taxa shared between the influent and WWTP to have a negative net growth rate (9). In these studies, it was concluded that this fraction of the immigrant community was inactive and did not contribute to the metabolism of the activated sludge. However, despite these findings, we empirically demonstrated that although displaying a negative net growth rate, active immigration-dependent genera are in fact growing and consuming substrates within the activated sludge as their abundance was higher than in the reactors receiving sterilized influent solids. This observation is in agreement with previous studies that demonstrated the rescue of complete nitrification in activated sludge bioreactors by nitrifiers in the influent stream (11). Consequently, observations of shared OTUs (ASVs or genera) do not accurately predict activity, and further work should be done to quantify the metabolic contribution of immigrants with net negative growth rates.

During Phase 2, continuous influent immigration prevented the competitive exclusion of the immigrating bacteria displaying low net growth rates, which were not well adapted to the reactor systems. This was confirmed during Phase 3 (reactor Sets B and C only), when reactors received synthetic wastewater only and immigration was removed. During this phase, a large proportion of immigration-dependent genera were washed out within 3 SRTs (Table 2). Of those remaining, between 75% and 77% exhibited a positive net growth rate during Phase 2. This result is consistent with reactor theory which describes that genera exhibiting a positive net growth rate should be maintained over time without the need for continuous immigration.

In contrast to the observed correlation between positive net growth rate and persistence through Phase 3, other immigrant-dependent genera were noted to display a positive net growth rate during Phase 2 but were not detected at the end of Phase 3 (Fig. 5). These counter observations could be due to a number of reasons. First, there may have been imprecisions and inaccuracies in the quantification of growth rates. This may be associated with the measurement of DNA yield or due to the general variability introduced by next-generation sequencing including through the rarefaction of data. Second, various abiotic and biotic phenomena likely impact the persistence of genera. Abiotic factors include the possibility that the influent solids themselves provided an additional food source that was removed during Phase 3. Biotic explanations may relate to the co-selection of immigration-dependent populations as a single unit, with some unit members exhibiting positive net growth rates and others exhibiting negative net growth rates (25). The reproducibility of the experimental system presented here would allow these different mechanisms to be explored in detail in future experiments.

The remainder of immigrants present at the end of Phase 3 displayed a negative overall net growth rate (Fig. 6). Generally, these taxa had a higher relative abundance (0.1% or above) at the end of Phase 2, whilst those with lower abundance were washed out by the end of Phase 3. A notable reduction (85% and above) in the abundance of these genera was observed within three SRTs, suggesting that washout would likely occur with prolonged reactor operation and no immigration.

### Resident core genera

Detailed studies of the microbial community composition of WWTP samples collected worldwide have enabled core taxa to be identified based upon their occurrence in different locations and their relative abundance (20). The core resident genera of the reactor communities overlapped with those previously reported in full-scale wastewater treatment plants. For example, some of the most abundant core resident genera, such as *Zoogloea*, *Haliangium*, and *Flavobacterium*, were previously classified as strict core genera, which were observed in at least 80% of the full-scale WWTPs surveyed with a relative abundance >0.1% (20). Whilst other abundant reactor core residents, such as *Aeromonas* and *Thermomonas*, were classified as “general core” taxa, which occurred in 50% of full-scale WWTP at an abundance of >0.1%. Taken together, these results demonstrate that the core community produced during this reactor study is representative of that in full-scale WWTPs.

### Growing immigrant genera

The population of immigrating bacteria varied based upon the source of influent solids received. Among reactor Sets A and B, the genera Spb280 and *Aquabacterium*, respectively (both family *Comamonadaceae*), were dominant immigrants (Fig. 4) and found exclusively within these reactor sets. Members of the *Comamonadaceae* family have previously been identified as habitat generalists capable of surviving in both influent wastewater and activated sludge (17). Thus, the presence of these immigrants is consistent with results from full-scale WWTP immigration studies.

Among reactor Set C, which received Pincourt influent solids, the genera in the immigrant population were more variable (Fig. 4). Whilst some abundant genera such as *Dechloromonas* appeared in all reactors, others such as *Elstera* and *Candidatus* Accumulimonas appeared more sporadically. This could be due to differences in niche availability in reactors or co-selection. Limitations associated with small-scale reactor experiments should also be considered. Given the influent solid particle size and the small reactor volume, there may have been a slight variability in the microbial community of the influent solids received by each reactor that exacerbated the observed community drifts.

Among the reactors, few examples of inoculum effect were observed. This validates this reactor protocol to be suitable for future immigration (or perpetual coalescence) studies, regardless of the activated sludge inoculum source. In reactor Set B, one example where inoculum appeared to have an impact was in the genus SipK9 (family *Xanthomonadaceae*), which was dominant only among reactors receiving inoculum A (La Prairie) and accounted for between 2.5% and 11.7% of overall reads. SipK9 is an uncultured genus previously reported in Antarctic mineral soils (26) and AS-WWTPs as documented in the MiDAS ecosystem-specific reference database (20). In the reactors inoculated with Pincourt and Cowansville activated sludge, the maximum abundance of SipK9 was 0.008% indicating that there were differences in niche availability or co-selection between the reactors based on the inoculum received. The function of this population is currently unknown. However, its high abundance warrants further investigation.

Control over specific populations may be key to both process optimization and mitigation of operational problems in activated sludge. In the immigrating community, specialized bacteria, such as the phosphorus-accumulating organisms *Dechloromonas*, *Candidatus* Accumulimonas, *Candidatus* Accumulibacter, and *Tetrasphera*, were identified (Fig. 4). Given the importance of these genera in enhanced biological phosphorous removal, immigration may be key in process optimization. Unfavorable taxa, such as *Gordonia*, a filamentous bacteria, and *Thauera*, which cause bulking and dewatering problems in activated sludge treatment plants, were also identified (27). By accounting for the degree of immigration in the design of solutions, operational problems in WWTPs could be managed more efficiently.

## MATERIALS AND METHODS

### Experimental design and reactor setup

In total, three ***sets*** of reactors were operated (Sets A, B, and C), which differed based upon the source of influent solids used. Figure 1b shows a basic schematic of *one* set of reactors operated. For the first set of reactors (Set A), the reactors received La Prairie (Québec, Canada) influent solids and were operated from May to September 2016. For the second and third sets (Sets B and C), the test reactors received Cowansville and Pincourt (Québec, Canada) influent solids, respectively, and were operated from May to November 2017. To investigate the impact of the starting community, each ***set*** of reactors was divided into three ***blocks*** (Blocks a, b, and c), the reactors in each block were inoculated with activated sludge obtained from either La Prairie, Cowansville, or Pincourt WWTPs, respectively (Fig. 1b).

The three wastewater treatment plants were selected as a source of influent solids and activated sludge inocula from among eight AS-WWTPs within a diameter of approximately 100 km from Montréal (Québec, Canada) based on the results of a previous study. In this study, it was revealed that the compositions of the activated sludge mixed liquor microbial communities at the three selected plants were at the extremes of the composition distribution when visualized using a principal coordinate analysis plot in a previous study (28). In other words, the activated sludge communities showed the greatest differences among the sites tested.

The reactors were operated in three phases (Fig. 1a), with the feeds altered to investigate the effect of immigration. During Phase 1, all reactors received Syntho (synthetic wastewater; defined in section *Feed Preparations*) only to develop a steady-state core microbial community adapted to the specific substrate composition. At the end of Phase 1, the activated sludge of all nine reactors that had received the same inoculum (i.e., 1 block of reactors; Fig. 1b) was combined and split again to ensure a homogeneous stable community in each reactor as described by Kaewpipat and Grady, 2002 (29). During Phase 2, the three test reactors in each inoculum block received Syntho supplemented with influent solids (resulting in nine test reactors per set) to simulate immigration into the AS. In addition, nine reactors (three per block) were defined as substrate controls and received Syntho supplemented with sterile influent solids (autoclaved at 121°C, 15 psi for 30 min). The final nine reactors (three per block) received Syntho only to act as a continuity control (nine per reactor set). In total, 72 reactors were operated comprising of three blocks of inoculum and three sources of influent solids, operated in triplicate resulting in 27 reactors per influent solids used. In 2017, there was only one set of Syntho-only controls used because Sets B (Cowansville influent solids) and C (Pincourt influent solids) were operated simultaneously, and their Syntho-only control reactors were equivalent (which allowed the economy of nine reactors). A detailed overview of the reactor experimental design is outlined in Table S2.

### Reactor operation

The reactors were inoculated with 90 mL of activated sludge mixed liquor (2.7 g-VSS/L) obtained from La Prairie (Block a), Cowansville (Block b), and Pincourt (Block c) WWTPs. The AS ML samples from the three WWTPs were collected within a period of 4 hours (Block a; May 2016, Block b and c; May 2017). Samples were transported on ice, stored at 4°C, and processed within 24 hours of collection. All reactors were incubated at 21°C with shaking at 180 rpm to allow aeration and gentle mixing. The reactors were operated with an SRT of 5 days which is within the range of typical values reported for conventional activated sludge treatment processes (30). The hydraulic retention time (HRT) for activated sludge processes typically ranges from 1.5 to 40 hours (30). In this study, due to the manual manipulation of reactors required, an average HRT of 1.8 days (36 hours) was selected. The HRT and SRT were controlled through daily feeding and wasting. Three times a week, the total reactor volume was settled in a 100 mL graduated cylinder for 45 min to allow solid-liquid separation to mimic the clarifier of an activated sludge wastewater treatment process, and the appropriate volume of the supernatant was removed.

### Influent solid processing for Phase 2 feed

Influent wastewater solids were concentrated at the WWTP by settling influent wastewater for 15 min in 20 L buckets. After settling, the supernatant was removed, and concentrated solids were transferred to 5 L containers. The concentrated influent wastewater was transported to the lab on ice and processed immediately. To separate the influent wastewater and solid fractions, the wastewater was centrifuged in 50 mL aliquots at 21,100 × *g* for 10 min (Thermofisher Scientific model ST16R Centrifuge). The supernatant was removed, and the solids were re-suspended in Synthetic wastewater to wash. The centrifugation and wash steps were repeated three times, and the influent solids were resuspended in a final ~800 mL of synthetic wastewater. The volatile suspended solids (VSS) concentration of the stock influent solid solution was measured using the Standard Method 2540E (31) before dilution. The concentrated influent solids stock was stored at 4°C for no more than 2 weeks before use.

### Feed preparation

A stock solution of influent solids was prepared as described in the section *Influent Solid Processing for Phase 2 Feed*. Synthetic wastewater “Syntho” was produced using a modified recipe as described by Boeije et al., 1999 (19). The main carbon sources in the feed included sodium acetate, dry meat extract, glycerol, starch, dry milk powder, linear alkylbenzene sulfonate (LAS), and Genapol X-080. The nitrogen sources included peptone, urea, uric acid, and ammonium chloride. The stock Syntho solution was autoclaved in 500 mL containers at 121°C and 15 psi for 30 min. Once diluted, the chemical oxygen demand (COD) of the Syntho feed was approximately 550 mg/L, and the total Kjeldahl nitrogen concentration was approximately 35 mg-N/L. The Syntho was supplemented with a trace element solution as described by Bollman et al., 2011, which contained the typical concentrations of elements found in wastewater (32). The “Syntho + Influent Solids” feed was prepared by diluting the stock influent solid solution in Syntho to a concentration of 120 mg-VSS/L, which represents the average VSS concentration of influent wastewater reported in the three wastewater treatment plants studied (33). The “Syntho + autoclaved solids” feed was prepared in the same way with the addition of autoclaving at 121°C/15 psi for 1 hour to sterilize.

### Sample analysis

Activated sludge reactor samples were collected weekly for analysis of total suspended solids and VSS using Standard Methods 2540B and 2540E respectively (31). Effluent wastewater (the supernatant after settling) samples were collected for the analysis of COD using Standard Methods 5220D (31). Biomass samples for DNA extraction were centrifuged and stored ready for nucleic acid extraction at −80°C until further use.

### 16S rRNA gene amplicon sequencing

DNA was extracted from stored biomass samples using DNeasy PowerSoil Kit (Qiagen, Germantown, MD, USA). PCR of the 16S rRNA gene V4 region was conducted using 515F (5’- GTGYCAGCMGCCGCGGTAA-3′) and 806R (5′-GGACTACNVGGGTWTCTAAT-3′) primers (34, 35). PCR conditions were as follows: 94°C for 3 min followed by 35 cycles of 94°C for 45 s, 50°C for 60 s, 72°C for 90 s, 72°C for 10 min, and a final hold at 4°C. Unique Uniprimer barcodes were added to the amplicons from each sample in a second PCR reaction to allow sample pooling. Reaction conditions were as follows: 94°C for 3 min followed by 15 cycles of 94°C for 30 s, 59°C for 20 s, 68°C for 45 s, 68°C for 5 min, and a final hold at 4°C. Barcoded amplicons were purified using QIAquick PCR Purification Kit (Qiagen, Germantown, MD, USA) and pooled at equimolar concentrations. Amplicons were sequenced on the Illumina MiSeq PE250 platform at McGill University and Génome Québec Innovation Center (Montréal, QC, Canada).

### Bioinformatic and statistical analyses

Sequencing data were analyzed using QIIME 2 (36) and R Software (packages “vegan” and “ape”). Specifically, the raw sequences were denoised and error corrected to produce quality-filtered reads and ASVs using DADA2 (37) in Qiime2 pipelines. Sequences were rarefied to 40,000 reads in R. ASV tables were exported directly from Qiime2 after quality filtering. Taxonomy was assigned using MiDAS 2.0 reference database (38) and tabulated. Microbial community diversity was analyzed using the “vegan” package of R (39) and Jaccard dissimilarity.

ANOSIM was conducted using the using the “vegan” package of R (40) to statistically test the significance of differences between groups. The ANOSIM test compared the mean of ranked dissimilarities between groups with the mean of ranked dissimilarity within groups (40).

An odds ratio test was used to assess whether the growth rate of immigrants (Fig. 5) impacted their fate during Phase 3 of reactor operation. Values were calculated by dividing the odds of the first group (genera with a positive net growth rate persisting at the end of Phase 3) by the odds of the second group (genera with a negative net growth rate persisting at the end of Phase 3).

Multiple sequence alignment was conducted using the amplicon sequencing variants for selected genera obtained using the DADA2 pipeline as described above. The multiple sequence alignment was performed using the BIONJ neighbor-joining algorithm.

### Definitions of population categories

The *core resident populations* were present in the majority of reactors and followed the criteria outlined by Wu et al. 2019 for core communities (7). Taxa were filtered based on the overall abundant genera by calculating the mean relative abundance of a given genera. Genera with a mean relative abundance of over 0.1% were selected as overall abundant taxa. Ubiquitous genera between reactors were identified based on their occurrence in over 80% of the reactor set (where *n* = 27; genera must have occurred in at least 22 reactors). Taxa that were present in reactors receiving either Syntho only or Syntho + influent solids but that appeared more sporadically (i.e., in fewer than 80% of reactors) or in lower abundance (below 0.1%) were classified as the *non-core resident populations*.

The populations of immigrating bacteria were determined and classified by comparing the reactors receiving influent solids with those receiving Syntho only (i.e., without active immigration). Bacteria present only in reactors receiving Syntho + influent solids (or sterile influent solids) were classified as influent immigrants, the presence of which was dependent upon immigration. Taxa occurring in both the reactors receiving influent solids and sterile influent solids were filtered based upon abundance; those occurring in equal abundance under both conditions were considered to be *residual immigrants* or residual DNA, whilst those occurring in higher abundance in the reactor receiving active solids were classified as *growing immigrants* (or *actively growing immigration-dependent populations*).

### Quantifying net growth rate with steady-state modeling

The impact of immigration on the average growth of taxa in activated sludge systems can be visualized using a scatter plot of their relative abundances in the activated sludge vs the influent wastewater. A steady-state mass balance on biomass and substrates can be used to develop a model quantifying the levels of immigration and the growth rate and mapping them on the relative abundance scatter plot. The model used here was partially developed by Grady et al., 2011 (41), and it is an extension of the model presented by Mei et al., 2019 (14). A detailed development is available in Guo et al., 2022, and its key elements are presented here for convenience (42).

With a mass balance on the biomass of the *i*-th taxon for an activated sludge system assuming completely mixed biomass, it is possible to show that the *i*-th taxon-specific growth rate ($\mu_{i}$) is given by eq 1.


(eq. 1) $$\mu_{i}=\left(\frac{1}{\theta_{x}}+b_{i}\right)\;\left(1-m_{i}\right)$$

Where $\theta_{x}$ is the solid retention time, $b_{i}$ is the decay rate of the *i*-th taxon, and $m_{i}$ is defined as the immigration level of the *i*-th taxon defined by eq. 2. The activated sludge biomass essentially results from two processes, immigration from the influent, and growth on the available substrates. Therefore, the immigration level is defined as the proportion of activated sludge biomass that immigrated from the inflow to the total activated sludge biomass.


(eq. 2) $$m_{i}=\frac{X_{Bio,i,Inf}}{X_{Bio,i,Inf}+Y_{i}S_{i,Inf}}=\frac{X_{Bio,i,Inf}}{X_{Bio,i,AS}\left(\theta /\theta_{x}\right)\;\left(1+b_{i}\theta_{x}\right)}$$

Where $X_{Bio,i,Inf}$ and $X_{Bio,i,AS}$ are the biomass concentrations of the *i*-th taxa in the influent and the activated sludge, respectively, $S_{i,Inf}$ is the concentration of substrates consumed by the *i*-th taxon, $Y_{i}$ is the biomass yield of the i-th taxon on the substrate consumed, and θ is the hydraulic retention time.

To map the growth rates and immigration levels of the relative abundance scatter plots, eq. 1 and eq. 2 can be developed considering the capture of the influent biomass of the *i*-th taxon by the activated sludge solids ($f_{OHO,Capt}$), the DNA extraction yields for the influent and activated sludge ($\gamma_{DNA,Inf},\;\gamma_{DNA,AS}$), and the total solids of the influent and the activated sludge ($X_{\;Tot,Inf},\;X_{\;Tot,AS}$). With these considerations, the relationship between the proportions of the *i*-th taxon in the influent and activated sludge as determined by 16S rRNA gene amplicon sequencing ($f_{16S,Inf,i},\;f_{16S,AS,i}$) is given by eq. 3.


(eq. 3) $$f_{16S,i,AS}=\frac{\theta_{x}}{\left(1-\mu_{net,i}\theta_{x}\right)}\cdot \frac{f_{OHO,Capt}}{\theta }\cdot \frac{X_{\;Tot,Inf}\gamma_{DNA,Inf}}{X_{\;Tot,AS}\gamma_{DNA,ML}}\cdot f_{16S,i,Inf}$$

Where $\mu_{net,i}$ is the net specific growth rate of the *i*-th taxon defined as specific growth rate minus specific decay rate ($\mu_{net,i}=\mu_{i}-b_{i}$). Eq. 3 demonstrates the mathematical relationship between the proportions of the *i*-th taxon in the influent and activated sludge, which is affected by the operational conditions of the reactor (HRT and SRT), and the net growth rate of the specific taxa. This equation has the advantage of providing a border representing a zero net growth rate in this study and a border of 100% immigration level in other studies.

The log-log version of eq. 3 (eq. 4) shows that taxa appearing on the same 45°-line in the log-log scatter plot of relative abundances have the same net growth rate and the same immigration level, and the y-intercept (bold term between square brackets) of this 45°-line is a function of $\mu_{net,i}$. Using Eq. 4, the border representing a zero net growth rate ($\mu_{net,i}=0$) can be drawn.


(eq. 4) $$\log\left(f_{16S,i,AS}\right)={\left[\log\left(\frac{\theta_{x}}{\theta }\cdot \frac{f_{OHO,Capt}X_{\;Tot,Inf}\gamma_{DNA,Inf}}{X_{\;Tot,AS}\gamma_{DNA,AS}}\right)-\log\left(1-\mu_{net,i}\theta_{x}\right)\right]}_{intercept}+1_{slope}\log\left(f_{16S,i,Inf}\right)$$

## Data Availability

The Illumina Miseq sequencing data from this study have been deposited to the European Nucleotide Archive under the project accession number PRJEB75447.

## SUPPLEMENTAL MATERIAL

Supplemental material aem.00598-24-s0001.pdf Tables S1 to S4; Figures S1 to S5.

ASM does not own the copyrights to Supplemental Material that may be linked to, or accessed through, an article. The authors have granted ASM a non-exclusive, world-wide license to publish the Supplemental Material files. Please contact the corresponding author directly for reuse.
