# Supplementary material for: Activated sludge microbial community assembly: the role of influent microbial community immigration
Source: Appl Environ Microbiol. 2024 Jul 12;90(8):e00598-24. doi: 10.1128/aem.00598-24 (PMC11337844; doi:10.1128/aem.00598-24)
Supplement: Supplemental material — Tables S1 to S4; Figures S1 to S5. [file aem.00598-24-s0001.pdf]

Activated Sludge Microbial Community Assembly: The Role of Influent Microbial Community Immigration

Claire Gibson[a]#, Shameem Jauffur[a]*, Bing Guo[a,b] & Dominic Frigon[a]

[a] Department of Civil Engineering and Applied Mechanics, McGill University, Montreal, Quebec, Canada.

[b] Centre for Environmental Health and Engineering, Department of Civil and Environmental Engineering, University of Surrey, United Kingdom

# Address correspondence to Dominic Frigon, Dominic.Frigon@mcgill.ca

* Present address: Environmental Department, Amazon Web Services, Canada

Table S1- Phases of reactor operation

Phase	Reactor Feed	Duration (SRTs) [1]	
		2016	2017
1	All receive Syntho only	12	10
2	Reactors receive three different feeds[2]	13	22
3	All receive Syntho Only	NA[3]	3

[1] One SRT represents 5 days of reactor operation

[2] Three feeds; Syntho and influent solids, Syntho and sterile solids and Syntho Only

[3] NA: not available; reactors in 2016 were not operated with a third phase

Table S2: Overview of the Distribution of Reactors in the Experimental Design (**Total Reactors:** 72)

	**Syntho + Influent Solids**			**Syntho + Sterile Solids**			**Syntho Only**		
	Block[1]			Block			Block		
Set[2]	**a**	**b**	**c**	**a**	**b**	**c**	**a**	**b**	**c**
**A**	3	3	3	3	3	3	3	3	3
**B**	3	3	3	3	3	3	3	3	3
**C**	3	3	3	3	3	3	Same as Set B[3]		

[1] Where Block a, b and c represent the different sources of the inoculum, where a- La Prairie activated sludge, b- Cowansville activated sludge, c- Pincourt activated sludge

[2] Where Set A, B and C represent the different sources of influent solids, where A- La Prairie influent, B- Cowansville influent, B- Pincourt influent

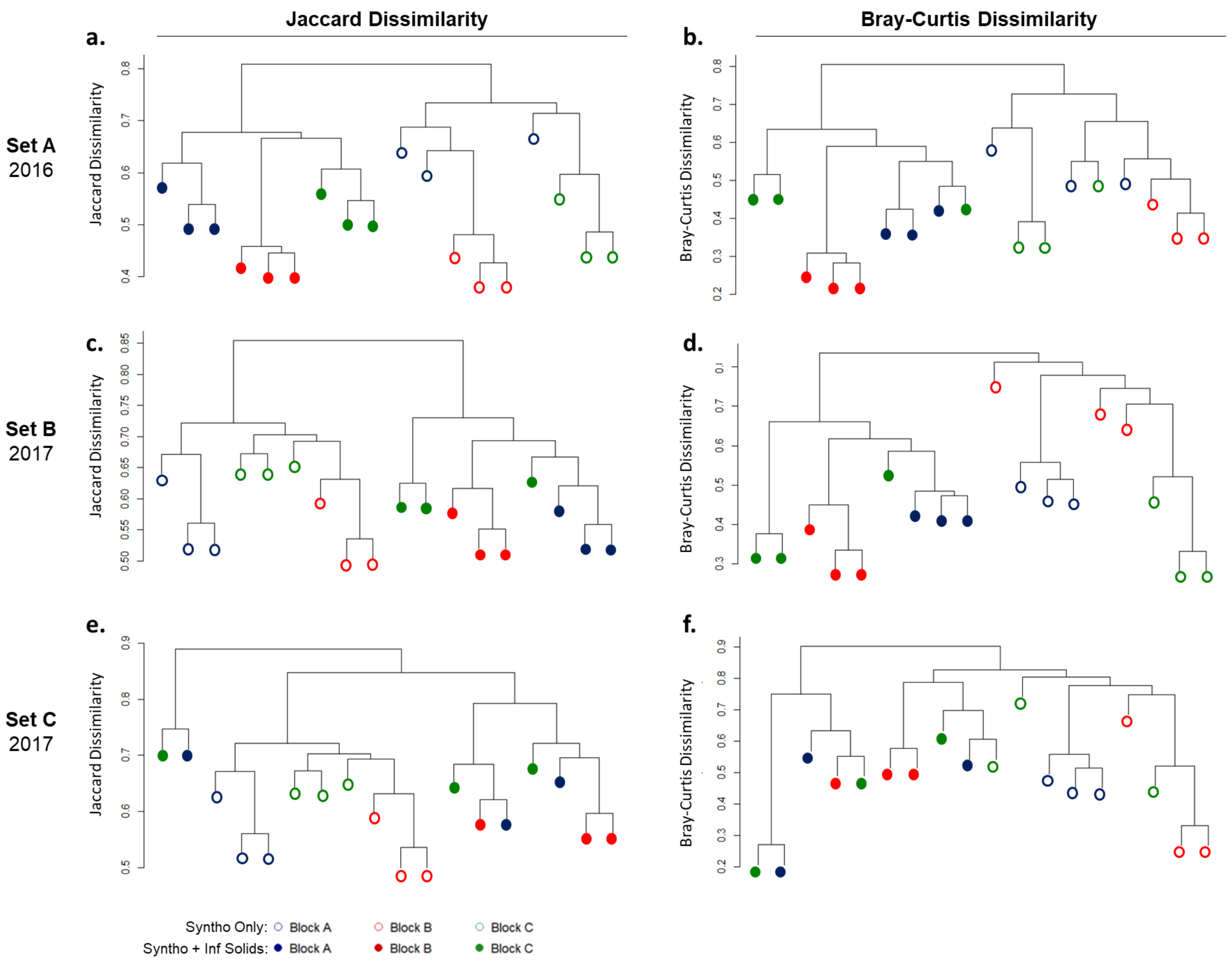

Figure S1- Tree dendrogram of microbial community at the end of Phase 2 using UPGMA clustering method and the Jaccard dissimilarity (a, c, e) or the Bray-Curtis dissimilarity (b, d, f). Filled symbols indicate test reactors which received influent solids during Phase 2. Hollow symbols indicate samples which received Syntho only throughout. Set A (a, b) were operated in 2016, whilst Sets B (c, d) and Set C (e, f) were operated in 2017. The Block indicates the source of inoculum: Block a-La Prairie mixed liquor, Block b-Cowansville mixed liquor, Block c-Pincourt mixed liquor. Inoculum communities were the same for Set B and C as they were operated in tandem.

Table S3- ANOSIM Analysis of Microbial Community at the End of Phase 2 (Figure S1)

		Jaccard Dissimilarity		Bray-Curtis Dissimilarity	
		ANOSIM R	α[1]	ANOSIM R	α
**Set A**	Syntho + Influent Solids	0.96	0.005	0.84	0.008
	Syntho Only	0.61	0.013	0.53	0.017
**Set B**	Syntho + Influent Solids	0.75	0.003	0.75	0.007
	Syntho Only	0.77	0.001	0.46	0.02
**Set C**	Syntho + Influent Solids	NS[2]	NS	NS	NS
	Syntho Only	0.77	0.003	0.46	0.017

[1] α – Significance level
[2] NS- Not significant

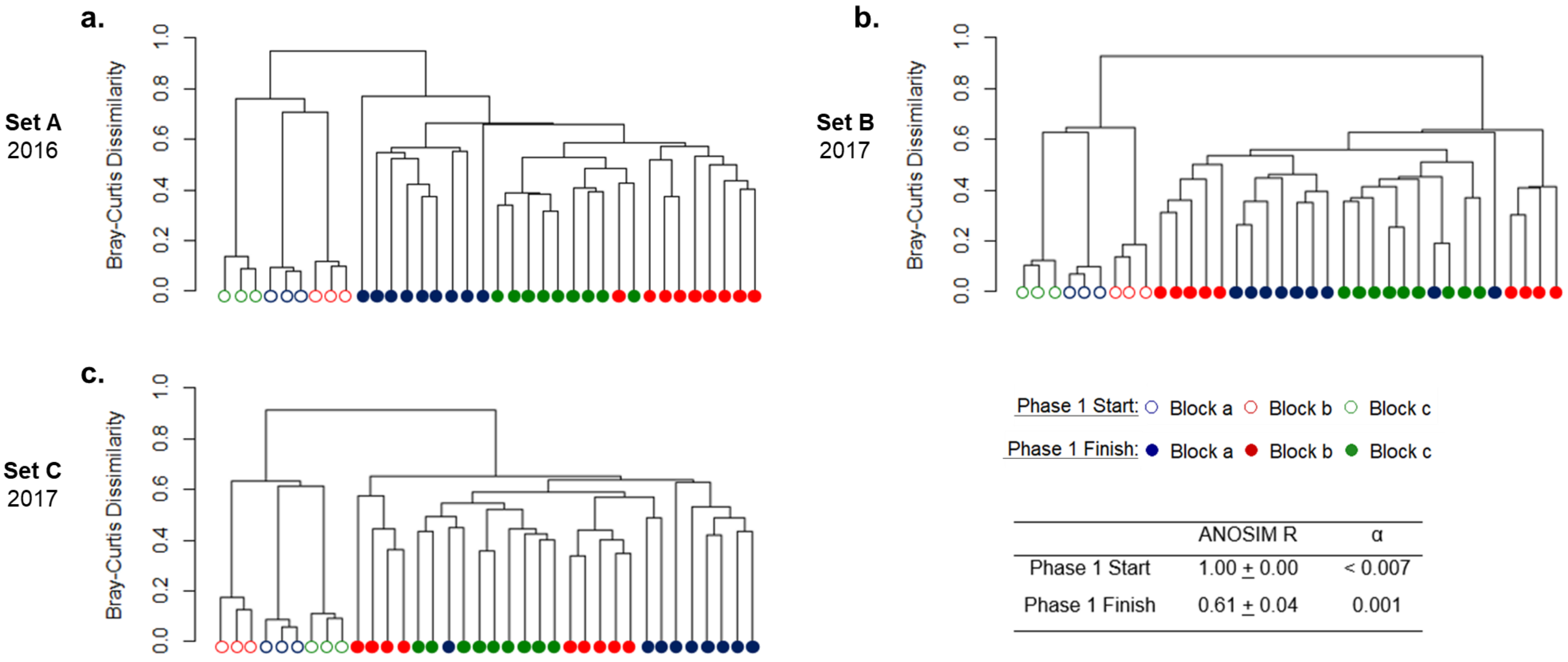

	ANOSIM R	α
Phase 1 Start	1.00 ± 0.00	< 0.007
Phase 1 Finish	0.61 ± 0.04	0.001

Figure S2- Tree dendrogram of microbial community at the start (hollow symbols) and end (filled symbols) of Phase 1 using UPGMA clustering method and the Bray-Curtis dissimilarity. Set A (a) were operated in 2016, whilst Sets B (b) and Set C (c) were operated in 2017. The Block indicates the source of inoculum: Block a- La Prairie activated sludge, Block b- Cowansville activated sludge, Block c- Pincourt activated sludge. Inoculum communities were the same for Set B and C as they were operated in tandem. Each inoculum (hollow symbols) represents a single biological sample that was extracted and sequenced 3 times, whilst the end of Phase 1 (filled symbols) are communities grown in independent reactors.

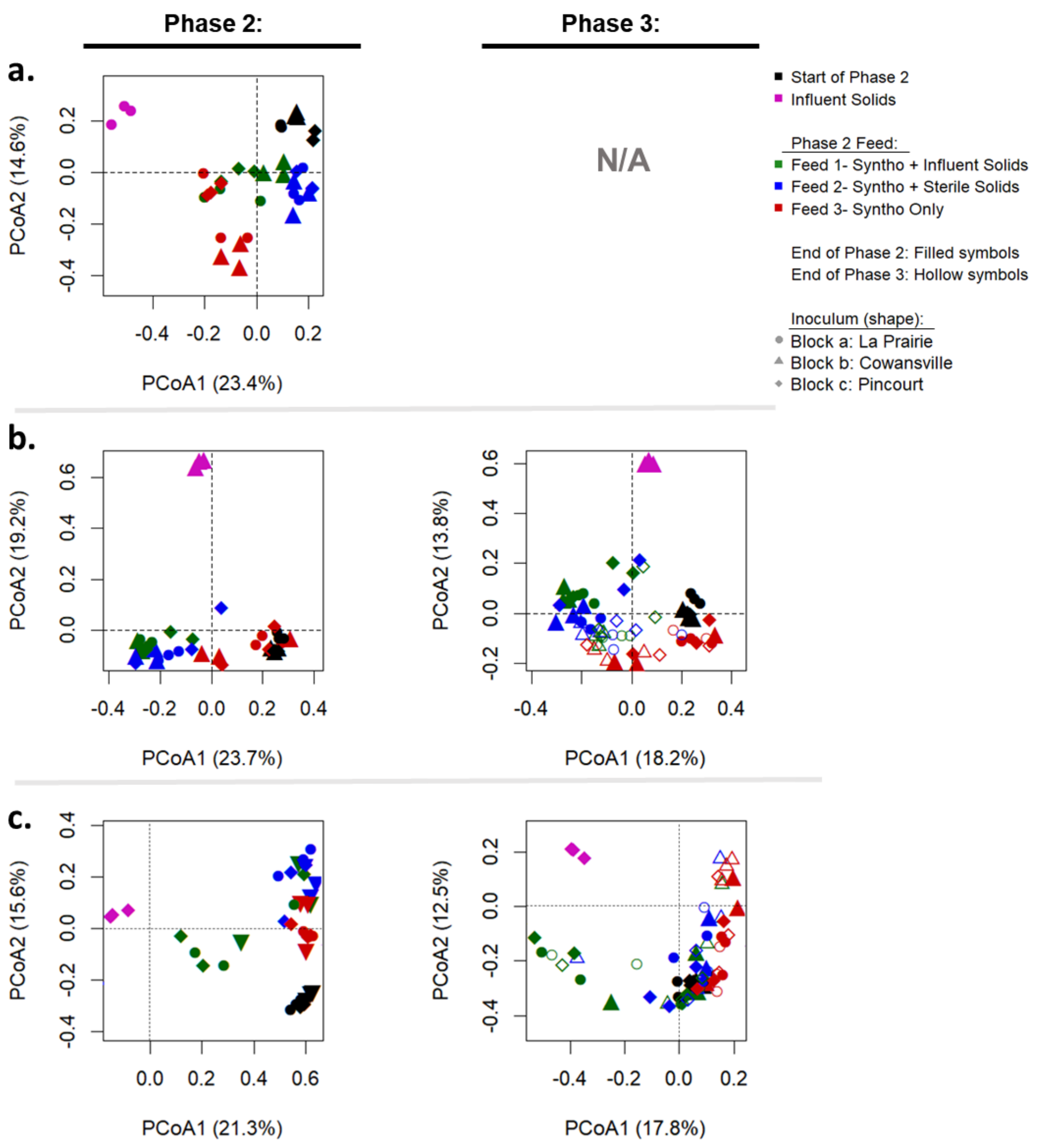

Figure S3- Principal coordinate analysis of reactors communities at the end of Phase 2 and 3, visualised at ASV level using Bray-Curtis Dissimilarity. a) Set A- La Prairie Influent Solids b) Set B- Cowansville influent solids c) Set C- Pincourt influent solids. Filled symbols represent samples taken at the end of Phase 2 and hollow symbols were samples collected at the end of Phase 3.

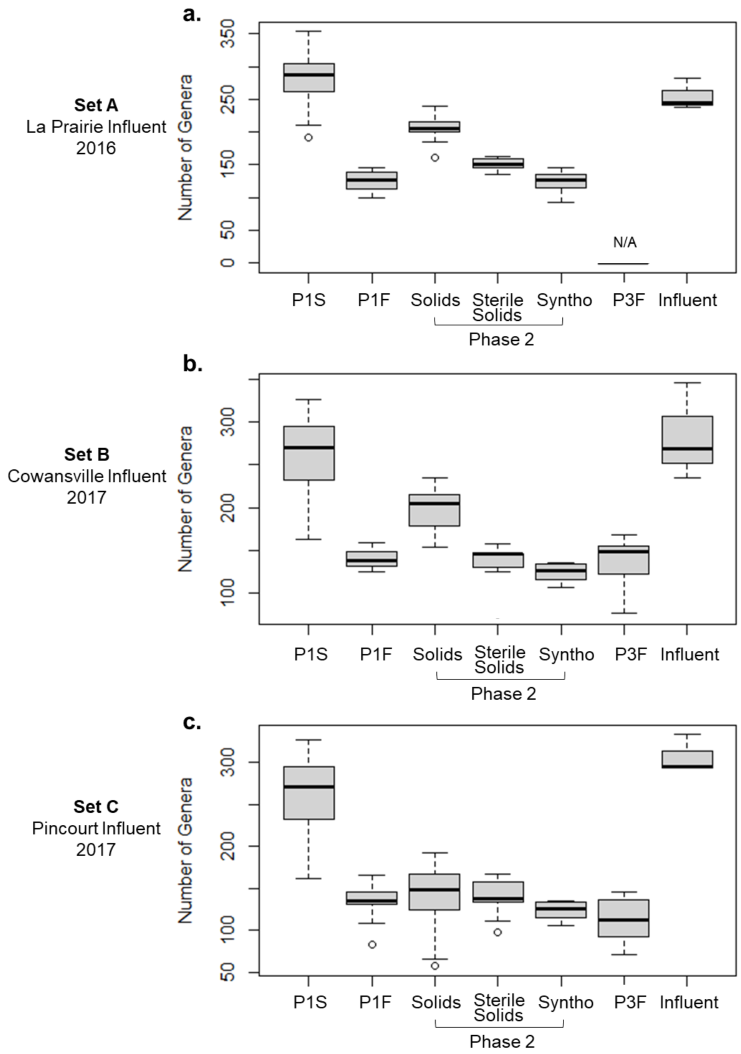

Figure S4- Genus richness boxplot of reactor communities in the inoculum at the beginning of the experiment (P1S; n = 9), at the end of Phase 1 (P1F; n = 27), end of Phase 2 (Solids: fed Syntho + influent solids, Sterile Solids: Syntho + autoclaved influent solids, and Syntho only; n = 9 each), end of Phase 3 for reactors receiving Syntho + influent solids (P3F; n = 9), and Influent Solids (n = 3). Phase 3 was completed for only reactor Sets A and B. a) Set A received La Prairie influent solids during Phase 2 b) Set B- Cowansville influent solids received c) Set C- Pincourt influent solids received. In the boxplot the middle bar represents the median number of genera within sample group. Box edges represent the first and third quartile. Whiskers represent the minimum and maximum number of genera.

Table S4: Values used in the calculation of the relationship between $f_{16S,i,ML}$, $f_{16S,i,inf}$, and $\mu_{net,i}$

**Parameter**	**Symbol**	**Reactor Set**		
		A- La Prairie	B- Cowansville	C- Pincourt
Solid Retention Time (days)	θ_x	5.0	5.0	5.0
Hydraulic Retention Time (days)	θ	1.8 ± 0.28	1.8 ± 0.28	1.8 ± 0.28
Fraction of influent captured by ML solids	$f_{OHO,Capt}$	1	1	1
Total VSS of the Influent (mg-VSS/L)	$X_{Tot,Inf}$	120	120	120
Total VSS of the ML (mg-VSS/L)[1]	$X_{Tot,ML}$	470.9[2]	685.3 ± 94.22	644.4 ± 73.54
DNA extraction yield of Influent (µg-DNA/mg-VSS)[3]	$\gamma_{DNA,Inf}$	1.73 ± 0.15	0.72 ± 0.07	0.94 ± 0.13
DNA extraction yield of ML (µg-DNA/mg-VSS)[4]	$\gamma_{DNA,ML}$	1.51 ± 0.09	1.75 ± 0.004	1.06 ± 0.09

[1]Where n=18 representing the average VSS of the nine reactors receiving influent solids (with immigration) over a two week period

[2]Calculated based upon COD/VSS ratio and validated with experimental data obtained during Phase 1 as no experimental measurements were obtained for this reactor set during Phase 2

[3]Influent DNA yield where n=3 and samples taken from three different time points

[4]Mixed liquor DNA yield where n=3 for each reactor set (1 reactor per block within each set). Samples obtained from the final day of operation

± indicates the standard deviation of reported measurements

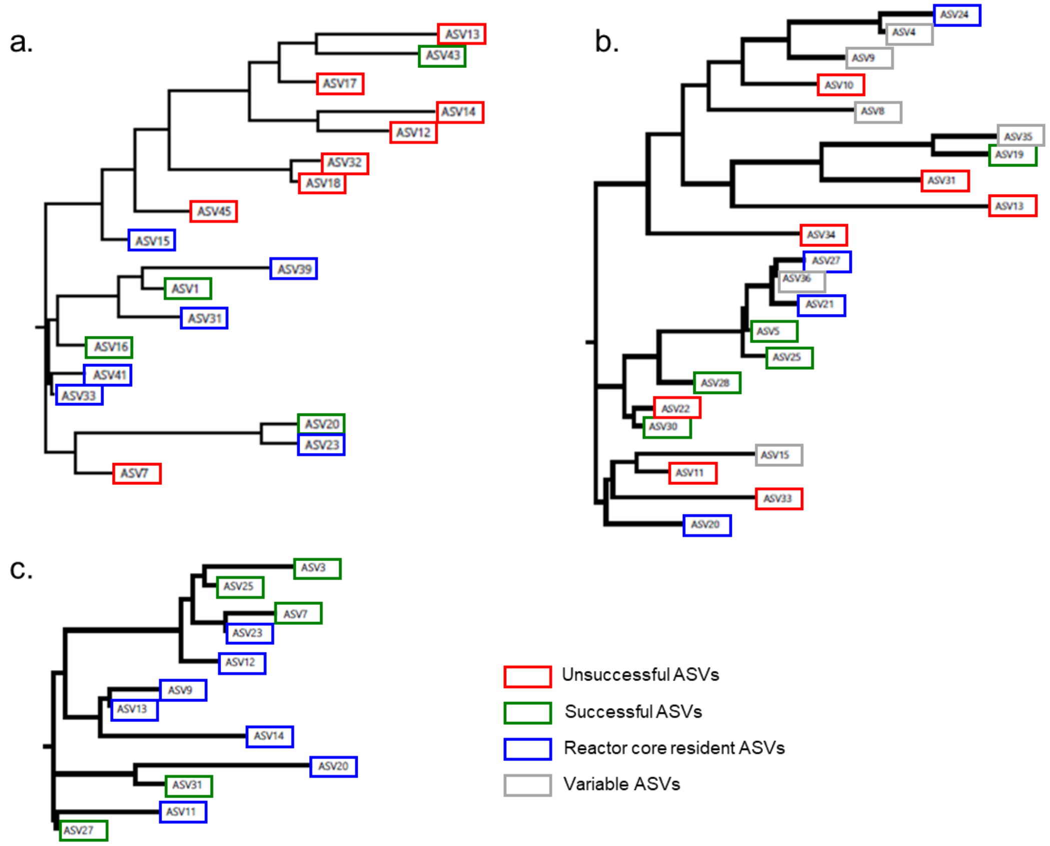

Figure S5- Multiple sequence alignment tree generated using BIONJ neighbour joining algorithm. a) Acinetobacter ASVs b) Pseudomonas ASVs c) Zoogloea ASVs. Unsuccessful ASVs are those that were detected in the influent but not in reactors with immigration. Successful ASVs are those detected in the influent and in the reactors with immigration only. Reactor core resident ASVs were those present in all reactors regardless of feed. Variable ASVs are those which had different fates in each reactor set (e.g. successful in one reactor set and unsuccessful in another).
